# Confidence Modulates the Conformity Behavior of the Investors and Neural Responses of Social Influence in Crowdfunding

**DOI:** 10.3389/fnhum.2021.766908

**Published:** 2021-11-04

**Authors:** Jiehui Zheng, Linfeng Hu, Lu Li, Qiang Shen, Lei Wang

**Affiliations:** ^1^School of Management, Zhejiang University, Hangzhou, China; ^2^Neuromanagement Laboratory, Zhejiang University, Hangzhou, China; ^3^School of Management, Zhejiang University of Technology, Hangzhou, China

**Keywords:** social influence, conformity behavior, confidence, crowdfunding, event-related potentials (ERP), feedback-related negativity (FRN), late positive potential (LPP)

## Abstract

The decision about whether to invest can be affected by the choices or opinions of others known as a form of social influence. People make decisions with fluctuating confidence, which plays an important role in the decision process. However, it remains a fair amount of confusion regarding the effect of confidence on the social influence as well as the underlying neural mechanism. The current study applied a willingness-to-invest task with the event-related potentials method to examine the behavioral and neural manifestations of social influence and its interaction with confidence in the context of crowdfunding investment. The behavioral results demonstrate that the conformity tendency of the people increased when their willingness-to-invest deviated far from the group. Besides, when the people felt less confident about their initial judgment, they were more likely to follow the herd. In conjunction with the behavioral findings, the neural results of the social information processing indicate different susceptibilities to small and big conflicts between the own willingness of the people and the group, with small conflict evoked less negative feedback-related negativity (FRN) and more positive late positive potential (LPP). Moreover, confidence only modulated the later neural processing by eliciting larger LPP in the low confidence, implying more reliance on social information. These results corroborate previous findings regarding the conformity effect and its neural mechanism in investment decision and meanwhile extend the existing works of literature through providing behavioral and neural evidence to the effect of confidence on the social influence in the crowdfunding marketplace.

## Introduction

Many daily decision makings, such as purchase decision (Kuan et al., 2015; De Martino et al., 2017), investment decision (Zhang and Liu, 2012; Wang et al., 2019), diet decision (Nook and Zaki, 2015; Higgs and Thomas, 2016), can be influenced by the choice or opinion of someone else. The consensuses of actions or opinions derived from the group are regarded as the social information that has been verified to play a vital role in guiding our behavior changes in line with the group, which is the manifestation of social influence (Cialdini and Goldstein, 2004; Cascio et al., 2015). Social influence has been found to be ubiquitous in several decision scenarios, such as perceptual decision (Chen et al., 2012; Germar et al., 2014), value-based decision (Zaki et al., 2011; De Martino et al., 2017), prosocial decision (van Hoorn et al., 2016; Wei et al., 2019), and its common consequence is to drive the conformity behavior, which means that the people change their behaviors to match it with the majority (Cialdini and Goldstein, 2004; Toelch and Dolan, 2015). Recently, the social influence of online investment has engaged attention from many researchers since the transaction platforms have embedded several functions to disclose social information such as the display of choices of the other investors or the fund-raising progress (Herzenstein et al., 2011; Lee and Lee, 2012). Given that the online environment is full of risk and information asymmetry, investors are inclined to look for more external cues to help themselves make “right” choices (Berkovich, 2011; Zhang and Liu, 2012). Therefore, the easily accessed social information, i.e., an investment decision of the others might lead to a stronger effect of the social influence in the online setting. Previous empirical studies have found that online investment decisions of the individuals indeed are influenced by the choices of other investors according to the evidence derived from the historical transaction data of the online platforms (Berkovich, 2011; Herzenstein et al., 2011; Lee and Lee, 2012; Yum et al., 2012; Zhang and Chen, 2017). A general phenomenon is that investors are more likely to invest in a project that has reached more transactions. For example, some studies analyze the panel data from online P2P loan websites to verify that the lenders have more likelihood to bid on an auction with more cumulative bids (Herzenstein et al., 2011; Zhang and Chen, 2017). These model studies interpret this conformity effect as investors believe that the others have additional valuable information, such as the credit ratings of the borrowers are higher than what the website describes, which helps them to make better investment decisions (Berkovich, 2011; Yum et al., 2012; Zhang and Liu, 2012).

Although prior studies have applied different models with the social information factors to confirm the social influence indeed exists in an online investment decision, they cannot directly reveal the underlying mechanisms including psychological and neural processes that lead to the conformity behaviors. Besides, individuals may vary in their conformity behaviors. In other words, people do not always consistently follow suit. They may insist on their own choices independently of the choices or opinions of others and even exhibit anticonformity behaviors, i.e., contradict the behaviors of the groups (Effinger and Polborn, 2001; Levy, 2004; Chen et al., 2010b; Wang et al., 2017). For example, Wang et al. (2017) find participants sometimes do not buy the stock that has been bought by more people or buy the stock even its buying power is lower. The authors speculate that individuals making antiherd decisions might be more confident in their ability and choices (Wang et al., 2017). Why does not social influence always work? Several studies have identified some contextual and personal factors modulating the social influence, such as task difficulty, group size, sex, and personality (Bond, 2005; Rosander and Eriksson, 2012; Wijenayake et al., 2020). In this study, we aim to uncover the essence of the varied susceptibilities to social influence from the perspective of confidence in the individual decisions, i.e., we would explore whether the confidence in initial decision influences the processing of the social information of an individual, and the following conformity behaviors.

As we know, uncertainty is inherent in decision making, and it can be reflected in the confidence of the individuals about their choices, which may fluctuate over time (De Martino et al., 2013; Ma and Jazayeri, 2014; Pouget et al., 2016; Schustek et al., 2019). Confidence refers to the belief regarding whether a decision is correct based on the available evidence (Ma and Jazayeri, 2014; Pouget et al., 2016). Many previous studies have confirmed that confidence is an essential component of decision making, and has impacts on the decision process of the people through revealing the relationships between confidence, accuracy, reaction time, and value perception (De Martino et al., 2013; Grimaldi et al., 2015; Dotan et al., 2018). More studies further explore the role that confidence plays in the decision based on neural evidence. For example, in a value-based task with the fMRI method, the authors find that decision confidence indeed takes part in the value comparison process and confidence information is integrated into the value presentation in the ventromedial prefrontal cortex (vmPFC) (De Martino et al., 2013). In addition to value-based decisions, the role of confidence in the perceptual decision has also been verified that confidence can track the evolution of the decision process and be computed continuously during this process (Gherman and Philiastides, 2015; Grimaldi et al., 2015). The studies mentioned earlier mainly focus on the role of confidence in the decision process itself, and other studies are also interested in the effect of confidence in the post-decision process. For example, van den Berg et al. (2016) found that in the two-sequential perceptual tasks, the confidence level in the previous decision task proportionally affects the speed accuracy trade-off of the participants in the second task. Besides, another study demonstrates that the participants with low confidence are more likely to seek additional information before making the final decision (Desender et al., 2018). Given that the effect of confidence has been investigated exclusively with the value-based or perceptual decision-making tasks, it remains a fair amount of confusion regarding the mechanism of how confidence plays a role in social conformity.

Some studies have provided primary evidence regarding the role of confidence in the conformity behaviors of the people. For example, both Morgan et al. (2012) and Cross et al. (2017) found that the confidence reported by the participants in the mental rotation task influence the answer-switch behaviors after seeing the response of the group, with the low-confident participants more likely to change their initial answers. This is also the case in an online quiz with the multiple-choice questions, in which the participants answered different questions and meanwhile reported their confidence (Wijenayake et al., 2020). The results show that the participants tend to conform to the answers of the groups when they are not sure of their answers (Wijenayake et al., 2020). Besides, De Martino et al. (2017) observe that belief of the people updating the product preference is adopt in a Bayesian fashion, which depends on both the reliability of the opinion of the group and the confidence of their own initial beliefs. Specifically, if the initial confidence of the people about their preference rating is low, they change more rating in the direction of the group rating, especially when the group rating is more reliable. However, it is still unclear to what extent the results emerging from the earlier studies can apply to the investment decision setting. To our knowledge, there are few studies directly investigating the confidence effect on the conformity behaviors in the investment decision as well as the underlying neural mechanism. The relationship between the effect of the social influence and decision confidence remains, so far, undetermined.

Neural mechanisms associated with the social influence have been widely studied, especially examining the core processes in response to the social information, i.e., the behaviors or opinions of the group (Schnuerch and Gibbons, 2014; Cascio et al., 2015; Toelch and Dolan, 2015). A classic paradigm used in many conformity-related neuroscience studies is the two-stages attractiveness rating task that originates from Klucharev et al. (2009). In detail, at the first stage, the participants are instructed to rate the attractiveness of the different faces, followed by showing the average rating of the group with fMRI measured continuously. Then, after a while begun the unannounced second stage, at which participants rate the attractiveness of the same faces again. Generally, the presented group rating serves as the social information eliciting the social influence and indeed affects the next rating adjustment, i.e., participants change their rating toward the average rating of the group. At the neural level, the conflict with the group rating activates the brain regions including rostral cingulate zone and the ventral striatum. Moreover, the amplitude of the activity in the ventral striatum can predict the following conformity behavior (Klucharev et al., 2009). Through applying this template of the experimental task, many studies reveal the neural correlates of conformity effect in various scenarios, such as music preference (Berns et al., 2010), product preference (De Martino et al., 2017), and willingness to invest (Wang et al., 2019). According to the results from diverse social conformity tasks with the fMRI method, two core processes consisting of conflict detection and valuation during the response to the social information are identified (Schnuerch and Gibbons, 2014; Cascio et al., 2015). In addition to fMRI results, other studies apply the event-related potential (ERP) method to provide electrophysiological evidence to the neural mechanism of the social influence. Many previous studies consistently find an early frontal negative component, e.g., feedback-related negativity (FRN), and a later parietal positive component, e.g., late positive potential (LPP), responding to the social deviation from the group (Chen et al., 2012; Kim et al., 2012; Shestakova et al., 2013; Schnuerch and Gibbons, 2015; Wang et al., 2019). FRN is a negative ERP component appearing around 200–300 ms at the frontal region after the onset of outcome and localizes in the anterior cingulate cortex (ACC) (Gehring and Willoughby, 2002; Holroyd et al., 2002; Nieuwenhuis et al., 2004). It has been shown as a neural signature of the prediction error of behavioral results of the people deviating from what they expect, indicating its role in performance monitoring, e.g., conflict/error detection (Kim et al., 2012; San Martín, 2012; Hauser et al., 2014; Ullsperger et al., 2014). Many social influence studies have indicated that more negative FRN is elicited by detecting the deviation from the group, such as the conflict between individual attractiveness ratings and the rating of the group (Shestakova et al., 2013; Schnuerch and Gibbons, 2015). LPP is a sustained positive ERP component typically beginning about 300 ms after stimuli onset and distributes mainly at the parietal region (Hajcak and Foti, 2020). Prior studies have explored the later stages of stimuli processing by capturing the deflections of LPP, which is modulated by the stimuli motivational significance (Hajcak et al., 2006, 2013; Thiruchselvam et al., 2016; Ma et al., 2017; Lin et al., 2018). In several ERP studies about social influence, larger LPP amplitude is observed for no conflict or small conflict between own opinion or behavior of the individual and the group (Kim et al., 2012; Schnuerch and Gibbons, 2015; Schnuerch et al., 2016; Thiruchselvam et al., 2017). All in all, the observed early and late ERP responses are consistent with the proposition about the two processes of conflict detection and valuation in the social influence.

To explore how confidence influences the trend to conformity behaviors of the individuals in investment decision and its neural mechanism, this study modified the experiment paradigm initiated by Klucharev et al. (2009) and set the context of crowdfunding, which is becoming a burgeoning and vital marketplace of the online investments (Agrawal et al., 2015; Genevsky et al., 2017; Wang et al., 2019). We think that uncovering the relationship between confidence and social influence in the crowdfunding market is of value both theoretically and practically. In detail, we designed a willingness-to-invest task consisting of the two stages with electroencephalogram (EEG) recording simultaneously. At the first investment stage, the participants observed a series of crowdfunding projects with brief information and evaluated the willingness they would invest. Then they need to declare how confident they felt about that decision. Finally, social information, i.e., willingness-to-invest rating of the group, was shown to the participants. At the unannounced second stage, the participants were instructed to observe the same projects and evaluated the willingness to invest and confidence again, without their initial rating and social information. This two-stage design can enable us to measure the neural response to the social information at the first stage, and identify the subsequent conformity effect by comparing the willingness rating at the second stage to that at the first stage (Klucharev et al., 2009). As with the previous studies, at the behavioral level, we revealed the effect of confidence on conformity behaviors by focusing on the changes of initial willingness to invest toward the group. Besides, regarding the neural mechanism, we aimed to clarify whether the confidence affects the perception of social information, both at the conflict detection and value evaluation stages, and focused on the manifestations of the two ERP components including FRN and LPP.

## Materials and Methods

### Participants

Thirty students from the Zhejiang University were recruited in this experiment, including 24 men and six women (*M*_age_ = 22.73 years, SE_age_ = 2.21 years). All the participants were right-handers with normal or corrected-to-normal vision, and none reported any history of mental diseases. This study was approved by the Ethics Committee of the Neuromanagement Laboratory in the Zhejiang University. All the participants signed the informed consent before the experiment started. Data of the three participants were rejected because of excessive recording artifacts. Another data from one participant was excluded because of an extreme willingness to invest. Finally, 26 participants were used in the data analysis.

### Stimuli and Procedure

**FIGURE 1:**
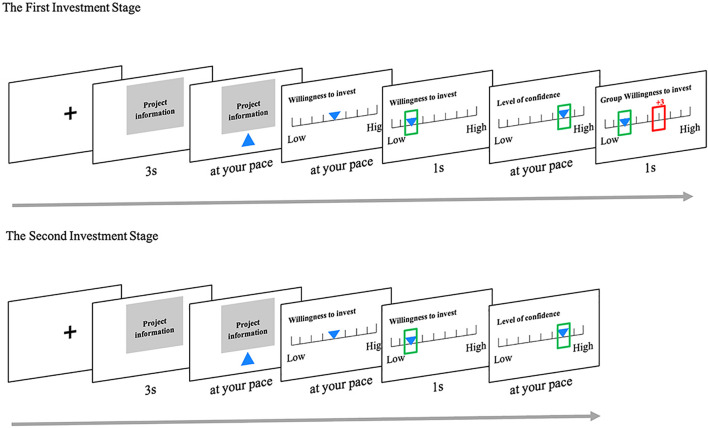
At the first stage, participants were instructed to learn the crowdfunding project for at least 3s (Participants could not go to the next page until the blue triangle appeared under the project picture) and then rated their willingness to invest this project as well as the confidence level about the decision by moving the cursor (a blue inverted triangle). Their ratings were highlighted by a green frame. At last, the group rating of willingness-to-invest was presented by a red frame with the rating difference from the participants above it. At the second stage, participants would rate the willingness-to-invest of the same projects and the corresponding confidence again.

All the stimuli in our study were extracted from a Chinese crowdfunding website, named JD Finance. We chose 100 pictures of the profit projects online and only retained the kernel information as regards the project content, viz. the title and the descriptive image of each project. The pictures were then processed with photoshop to the same size of 790 × 400 pixels. We employed a variant version of the rating task initially developed by Klucharev et al. (2009) (Figure 1). The task was run by python 2.7.

This task contained two investment stages. At the first stage, participants were instructed to rate the willingness to invest in the 100 projects one by one in a random order as well as the confidence about each decision. In detail, each trial started with a fixation lasting for 1,000 ms to remind participants to focus, and a new trial has begun. Then, a crowdfunding project picture was mandatorily presented for 3 s during which the participants were instructed to learn the project carefully and not make a response. After 3 s, a blue triangle appeared under the project picture, indicating that the participants could press number 3 to continue to the willingness-rating page at their pace. At the willingness-rating page, the participants press “1” (left direction) or “3” (right direction) to move the cursor (a blue inverted triangle) to rate their willingness to invest in the project from 1 to 8 (1 – the lowest willingness to 8 – the highest willingness). Once they had decided, they pressed “2” and a green frame around their choice was highlighted for 1 s for confirmation. Then the confidence-rating page showed up and the participants need to rate their confidence level about the decision just made from 1 to 7 (1 – the lowest confidence to 8 – the highest confidence) as the way in the willingness-rating page. Finally, they were shown willingness ratings from both the group and their own for 1 s. The rating of the group was highlighted by the red frame with the rating difference from the participants above it. These 100 trials at the first stage were divided into two blocks. The second investment stage that was unannounced before started about 20 min after finishing the first-stage task. The experiment procedure at the second stage was similar to that of the first stage in which the participants require to rate the willingness to invest in the same 100 crowdfunding projects again and the confidence levels of each decision. But the difference was that there were not any group ratings displayed to the participants.

Before the experiment, the rating of the group was announced to each participant as an average rating collected from another 50 participants in our previous behavior experiment. The group ratings were manipulated based on the rating of the participants to induce conflict between the group and the behaviors of the participants. Specifically, we manipulated the differences between the rating of the group and an initial rating of the participants to be −3, −2, −1, 1, 2, and 3. Among the 60% of all 100 trials, the ratio of each different type of −3, −1, 1, and 3 was kept approximately equal for each participant by using an adaptive algorithm. The remaining 40 trials included 10 trials of ± 2 difference type and 30 trials of 0 difference type (no conflict), which served as fill trials and were excluded in the final analysis. In sum, there were four main conflict conditions including 2 magnitudes (small: −1, 1 vs. big: −3, 3) × 2 valences (positive: 3, 1 vs. negative: −3, −1).

Before the formal experiment, five practice trials were provided to make the participants familiar with the procedure. All the participants were seated comfortably in front of the screen in a professional EEG experiment room, which was dimly lit, sound-attenuated, and electrically shielded. At the end of the experiment, they were paid 70 RMB as compensation, and no one was suspicious of the facticity of group rating.

### Electroencephalogram Recording

During the experiment, we used 64 scalp sites with a Neuroscan Synamp2 Amplifier (Scan4.3, Neurosoft Labs Inc., Sterling, VA, United States) to record EEGs (band-pass 0.05 Hz to 70 Hz, sampling rate 500 Hz), with the electrodes on the cephalic region as the ground and the left mastoid as an online reference. Besides, supra- and infra-orbital electrodes on the left eye were used to record vertical electrooculogram (EOG), and electrodes on the outer canthi of both the eyes recoded the horizontal EOG. The electrode impedance was maintained below 5 kΩ in the whole experiment.

### Data Analysis

In our study, the social information was the average group rating of the willingness to invest in a specific crowdfunding project. The conflicts between the social information and the self-information of the participants (self-rating) were manipulated within each participant, divided into four types of −3, −1, 1, and 3. We mainly focused on how the rating of the participants changed at the second stage along with these conflict types, and what were the neural responses to these conflicts during the revelation of the social information.

#### Behavioral Data

For behavioral data, we calculated two indexes to present the influence of social information, including conformity behavior change and conformity rate. Conformity behavior change was defined as the extent to which rating of the participants changed along with the group rating when the conflict existed. This change in the conformity behavior equaled to the difference between final willingness to invest (rating at the second stage) and initial willingness to invest (rating at the first stage), i.e., conformity behavior change = final rating - initial rating. We defined the conformity trials in which the participants changed the rating in the direction of the group rating and the conflict trials in which the initial rating of the willingness of the participants to invest at the first stage were different from the rating of the group. Thus, the conformity rate was calculated as the percentage of the conformity trials in all the conflict trials for each participant, i.e., conformity rate = number of conformity trials/number of conflict trials. What is more, the confidence level of each trial was divided into the low and high conditions according to the average confidence rating of each participant of the total 100 trials. Specifically, trials with the confidence rating below average rating were categorized as low-confidence conditions while trials with the confidence rating above average rating were categorized as high-confidence conditions. The within-participant repeated-measures ANOVA was conducted on the absolute value of conformity behavior change and conformity rate, with the factors of conflict magnitude (small: −1, 1 vs. big: −3, 3), valence (positive: 3, 1 vs. negative: −3, −1), and confidence (low vs. high).

#### Electroencephalogram Data

All the EEG data were analyzed offline, with re-referenced to the average of the left and right mastoids and the ocular artifacts removed. We used a low pass of 30 Hz, 24 dB/octave to filter the recorded EEGs. The epoch began at −200 ms prior to group rating onset and ended at 1,000 ms after onset, with the prior 200 ms serving as a baseline. All the epochs that contained extreme amplitude (exceeding ± 80 μV) were excluded. For each participant, all the single-trial data were obtained averaged for the four conflict conditions consisting of 2 (conflict magnitude: small vs. big) × 2 (confidence: low vs. high). Specifically, we averaged the EEGs by the high confidence/high conflict, high confidence/low conflict, low confidence/high conflict, low confidence/low conflict. All these preprocessing were conducted by the EEGLAB in MATLAB (Delorme and Makeig, 2004). At the stage of social information processing, we mainly focused on the FRN and LPP as in previous studies (Schnuerch and Gibbons, 2015). Based on the visual inspection and previous studies, FRN during 260 to 300 ms at the fronto-central area (F1, Fz, F2, FC1, FCz, and FC2) and LPP during 500–700 ms at the center-parietal area (CP1, CPz, CP2, P1, Pz, and P2) were chosen for the analysis.

All in all, the with-participant repeated measures ANOVAs on the mean amplitude of FRN with confidence (low, high) × conflict magnitude (small vs. big) × electrodes (F1, Fz, F2, FC1, FCz, and FC2), and LPP with the confidence (low, high) × conflict magnitude (small, big) × electrodes (CP1, CPz, CP2, P1, Pz, and P2) were conducted. Greenhouse–Geisser correction was also used when necessary.

## Results

### Behavioral Results

**FIGURE 2:**
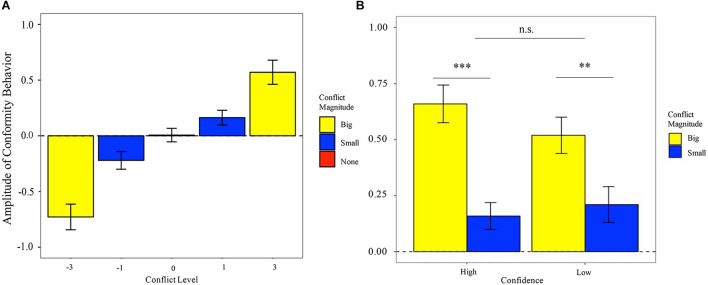
**(A)** Changes of willingness-to-invest (rating at the second stage – rating at the first stage) across the different conflict levels with the rating of the group. **(B)** Effect of confidence (low vs. high) × conflict magnitude (small vs. big) on absolute changes of willingness-to-invest. ^∗∗∗^*p* < 0.001, ^∗∗^*p* < 0.01.

*Conformity Behavior change*: As shown in Figure 2A, at the second stage, the participants changed their willingness-to-invest toward group ratings. The larger the conflict between individual and group rating was, the more change of the behavior was made by an individual. The 2 (valence: positive, negative) × 2 (magnitude: small, big) ANOVA result indicated that there was a prominent main effect of the magnitude [*F*_(1,25)_ = 29.781, *P* < 0.001, η^2^ = 0.544], but no significant effect of valence [*F*_(1,25)_ = 0.702, *P* = 0.41, η^2^ = 0.027] as well as the interaction effect between them [*F*_(1,25)_ = 0.349, *P* = 0.536, η^2^ = 0.016]. In detail, the conformity behavior changes in the big conflict condition (*M*_big_ = 0.649, SE_big_ = 0.066) were significantly larger than that in the small conflict condition (*M*_small_ = 0.192, SE_small_ = 0.044).

Given the null effect of conflict valence on the behavior adjustment, we combined the deviations with the same magnitude for further analysis. As shown in Figure 2B, the 2 (confidence: low, high) × 2 (magnitude: small, big) ANOVA result showed that the main effect of conflict magnitude [*F*_(1,25)_ = 23.803, *P* < 0.001, η^2^ = 0.488] was significant. However, the main effect of confidence [*F*_(1,25)_ = 0.637, *P* = 0.432, η^2^ = 0.025] and the interaction effect [*F*_(1,25)_ = 1.516, *P* = 0.230, η^2^ = 0.057] were not significant.

**FIGURE 3:**
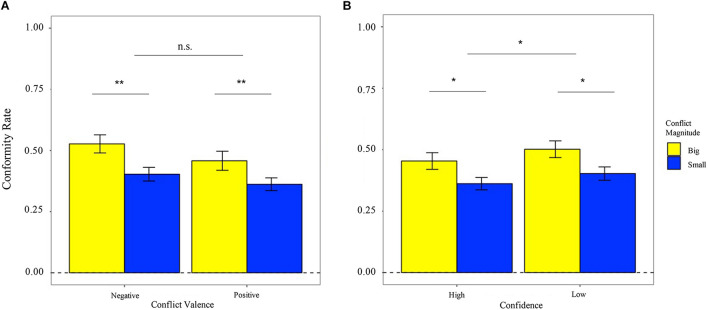
**(A)** Effects of conflict valence (negative vs. positive) × conflict magnitude (small vs. big) on conformity rate. **(B)** Effects of confidence (low vs. high) × conflict magnitude (small vs. big) on the conformity rate. **p < 0.01, *p < 0.05.

*Conformity rate*: As shown in Figure 3A, the 2 (valence: positive, negative) × 2 (magnitude: small, big) ANOVA result indicated that there was a prominent main effect of the magnitude [*F*_(1,25)_ = 12.010, *P* = 0.002, η^2^ = 0.325], but there was no significant effect of valence [*F*_(1,25)_ = 1.878, *P* = 0.183, η^2^ = 0.070] as well as the interaction effect between them [*F*_(1,25)_ = 0.390, *P* = 0.538, η^2^ = 0.015]. In detail, the conformity rate in big conflict condition (*M*_big_ = 49.2%, SE_big_ = 0.028) was significantly larger than that in the small conflict condition (*M*_small_ = 38.3%, SE_small_ = 0.019).

Similarly, we did not observe significant asymmetric effect of the valence on the conformity rate, and thus, we combined the deviations with the same magnitude. As presented in Figure 3B, the 2 (confidence: low, high) × 2 (conflict magnitude: small, big) ANOVA result showed that the main effects of confidence [*F*_(1,25)_ = 5.079, *P* = 0.033, η^2^ = 0.169] and conflict magnitude [*F*_(1,25)_ = 9.828, *P* = 0.004, η^2^ = 0.282] were both significant. However, the interaction effect was not significant [*F*_(1,25)_ = 0.012, *P* = 0.912, η^2^ < 0.001]. Specifically, the conformity rate in high-confidence condition (*M*_high_ = 40.8%, SE_high_ = 0.021) was significantly lower than that in the low-confidence condition (*M*_low_ = 45.3%, SE_low_ = 0.022).

### Electroencephalogram Results

#### Feedback-Related Negativity

**FIGURE 4:**
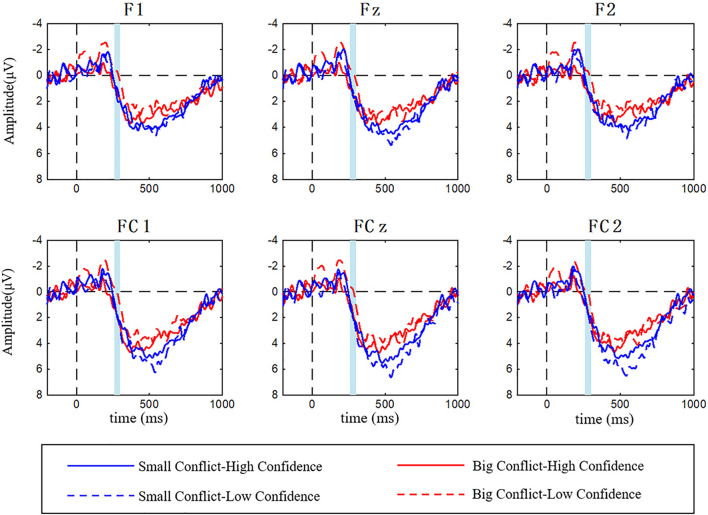
Grand averaged ERPs elicited by confidence (low vs. high) × conflict magnitude (small vs. big) with FRN (the shaded area from 260 to 300 ms) at F1, Fz, F2, FC1, FCz, and FC2.

Event-related potential waveforms with an FRN elicited during the evaluation of social information under interaction of conflict magnitude and confidence at six electrodes of F1, Fz, F2, FC1, FCz, and FC2 were shown in Figure 4. The ANOVA results showed that the main effect of conflict magnitude was significant [*F*_(1,25)_ = 7.049, *P* = 0.014, η^2^ = 0.220], while the main effects of confidence [*F*_(1,25)_ = 3.448, *P* = 0.075, η^2^ = 0.121], and electrode [*F*_(5,125)_ = 1.668, *P* = 0.147, η^2^ = 0.063] were not significant. Big conflict elicited more negative FRN amplitude as compared to the small conflict (M_big–conflict_ = 0.267 μV < M_small–conflict_ = 1.563 μV). All the interaction effects including conflict magnitude × confidence [*F*_(1,25)_ = 3.26, *P* = 0.083, η^2^ = 0.115], conflict magnitude × electrode [*F*_(5,125)_ = 1.758, *P* = 0.126, η^2^ = 0.066], confidence × electrode [*F*_(5,125)_ = 0.948, *P* = 0.453, η^2^ = 0.037] and conflict magnitude × confidence × electrode [*F*_(5,125)_ = 0.688, *P* = 0.633, η^2^ = 0.027] were not significant.

#### Late Positive Potential

**FIGURE 5:**
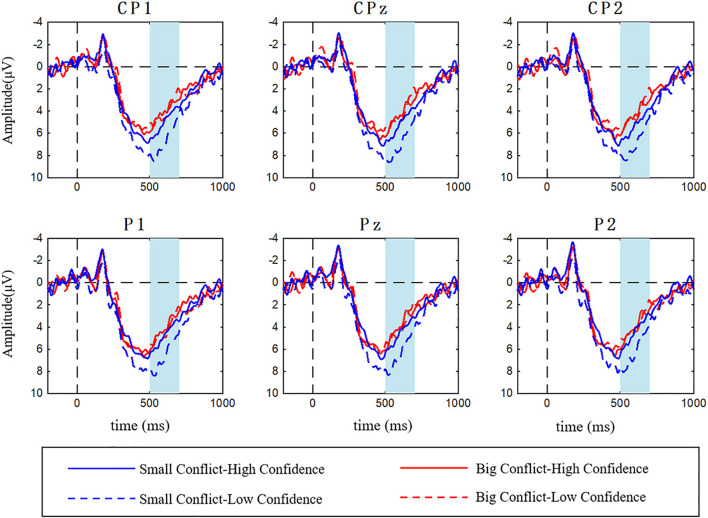
Grand averaged ERPs elicited by confidence (low vs. high) × conflict magnitude (small vs. big) with LPP (the shaded area from 500 to 700 ms) at CP1, CPz, CP2, P1, Pz, and P2.

Event-related potential waveforms with LPP elicited during the evaluation of the social information under interaction of the conflict magnitude and confidence at six electrodes of CP1, CPz, CP2, P1, Pz, and P2 were shown in Figure 5. The ANOVA results showed that the main effect of the conflict magnitude was significant [*F*_(1,25)_ = 17.318, *P* < 0.001, η^2^ = 0.409], the main effect of confidence was marginally significant [*F*_(1,25)_ = 4.008, *P* = 0.056, η^2^ = 0.138], but the main effect of electrode was not significant [*F*_(5,125)_ = 1.576, *P* = 0.172, η^2^ = 0.059]. Small conflict elicited more positive LPP amplitude than the large conflict (M_small–conflict_ = 5.490 μV > M_big–conflict_ = 4.002 μV). While the low confidence elicited more positive LPP amplitude than the high confidence (M_low–confidence_ = 5.102 μV > M_high–confidence_ = 4.390 μV). The interaction effect of the conflict magnitude and confidence was significant [*F*_(1,25)_ = 5.103, *P* = 0.033, η^2^ = 0.170]. We adopted a simple effect analysis and found that in the low confidence condition, small conflict evoked larger LPP amplitude than the big conflict (M_small–conflict_ = 6.263 μV, M_big–conflict_ = 3.941 μV, *P* = 0.001). But the conflict magnitude effect was not significant in the high confidence condition (M_small–conflict_ = 4.717 μV, M_big–conflict_ = 4.063 μV, *P* = 0.078). We did not observe the significant interaction effects of conflict magnitude and electrode [*F*_(5,125)_ = 1.175, *P* = 0.325, η^2^ = 0.045], confidence and electrode [*F*_(5,125)_ = 0.519, *P* = 0.761, η^2^ = 0.020] as well as conflict magnitude, confidence and electrode [*F*_(5,125)_ = 0.578, *P* = 0.717, η^2^ = 0.023].

## Discussion

This study aimed to uncover the social influence in the context of crowdfunding, and further figure out the modulatory effect of individual decision confidence on the social influence with both the behavioral and neural evidence. We applied a modified conformity task derived from the study by Klucharev et al. (2009). The behavioral effect of the social influence was focused on how the conformity behavior change (referring to what extent the rating at the second stage altered toward a rating of the group) and conformity rate (referring to the percentage of conformity trials in all the conflict trials) varied according to the magnitude of conflicts between willingness-to-invest of the participants and the group. As for the neural manifestations, we mainly characterized the neural responses to the social information by revealing the deflections of FRN and LPP elicited by the small vs. big conflicts. Finally, we examined the confidence effect on the conformity behaviors and neural perception of the social information. Our behavioral results demonstrate that the investment decision of the people indeed is influenced by the opinion of the others, i.e., when the people are aware of their rating different from that of the group, they are likely to change their rating in line with the group. Specifically, both the conformity behavior change and conformity rate were larger in the big conflict condition than those in the small conflict condition. By considering the confidence effect, only the conformity rate was higher in the low-confidence condition than that in the high-confidence condition, indicating the conformity effect is stronger when people experience high uncertainty. Regarding the neural mechanism, we observed that more negative FRN and decreased LPP amplitude were elicited by the big conflict than the small conflict. However, we only discovered the impact of the confidence on the LPP, which showed that the LPP was more positive for low confidence compared with the high confidence. Besides, the LPP difference between the small and big conflicts was more obvious in the low confidence than that in the high confidence, demonstrating confidence modulates the neural processing of the social information at the later stage. These ERP results provide evidence to account for the conformity behavior. That is small and big conflicts are evaluated differently in the brain, and therefore result in the different magnitude of conformity behaviors. In addition, low confidence makes people rely more on social information according to the larger LPP in the low-confidence condition. To the best of our knowledge, this study first provides insights into the confidence effect on the social influence in the crowdfunding investment and suggests that confidence can modulate neural sensitivity of the people to the social information during the investment decision and the following conformity behaviors.

Social influence is an important factor in the various investment decisions, especially in an online environment possessing accessible social information and high uncertainty. Our behavior results are consistent with the findings of the previous studies that the people are inclined to change their own opinions to follow the opinion of the group and this social influence effect becomes obvious along with conflict magnitude increases (Shestakova et al., 2013; Huang et al., 2014; Schnuerch and Gibbons, 2014, 2015; Wang et al., 2019). Besides, these results provide additional experimental evidence to the modeling results of conformity effect in online investment (Berkovich, 2011; Lee and Lee, 2012; Yum et al., 2012; Zhang and Liu, 2012; Zhang and Chen, 2017) and extend it to the crowdfunding context.

Confidence can be treated as a measurement of the subjective estimate regarding whether individuals make the right decision (Peterson and Pitz, 1988; Ma and Jazayeri, 2014; Pouget et al., 2016). Generally, the lower confidence individuals experience, the higher uncertainty they feel while making decisions (De Martino et al., 2013, 2017; Ma and Jazayeri, 2014; Meyniel et al., 2015; Pouget et al., 2016). In our study, the confidence was reported trial-by-trial and reflected the degree of the subjective uncertainty in making a correct willingness-to-invest of the crowdfunding projects. Several studies suggest that confidence not only plays a role in the evaluation of the decision process itself but also has an impact on the subsequent decision performance (van den Berg et al., 2016; Desender et al., 2018, 2019; Frömer et al., 2021). We found that confidence had a negative effect on the conformity rate, i.e., low confidence elicited more conformity behaviors. Our results of the confidence effect are consistent with the previous studies that indicate social influence effect appeared more obvious when decision-makers undergo low confidence (Morgan et al., 2012; Shestakova et al., 2013; Cross et al., 2017; De Martino et al., 2017). For example, Morgan et al. (2012) find that people are increasingly likely to switch to the decision of the conflicting group when their confidence in their own decision is low. Another study indicates that the preference of the people about the product is more likely to be changed when they hold low confidence in their initial preference judgments (De Martino et al., 2017). It is noticed that the aforementioned studies mainly apply psychological tasks, such as mental rotation, or value-based tasks. This study provides direct evidence to support the confidence which also works on the conformity behaviors in the crowdfunding decision. However, we fail to find the confidence effect on the levels of the behavior change, and we suggest this might be attributed to that albeit low-confident individuals who change their initial rating more likely, and they would not change to lower or more than the rating of the group. In other words, people who change the rating regardless of the confidence just aim to be consistent with the group.

At the neural level, we mainly uncover the neural mechanisms underlying the processing of social information. Previous neuroimage studies provide converging evidence that the performance-monitoring and reward-related brain regions participate in the social influence (Zaki et al., 2011; Izuma, 2013; Schnuerch and Gibbons, 2014; Cascio et al., 2015). Accordingly, some studies observe more ACC activation when people confront the opinion of a group that is against their own (Klucharev et al., 2009; Berns et al., 2010; Stallen et al., 2013; Park et al., 2017). As the ACC is an important brain region involved in conflict detection (Kerns et al., 2004), it is inferred that an early conflict detection exists during the evaluation of the social information (Schnuerch and Gibbons, 2014; Cascio et al., 2015; Toelch and Dolan, 2015). In addition to fMRI findings, some ERP studies document that the more negative FRN is elicited by the conflict opinion in various conformity tasks (Chen et al., 2012; Kim et al., 2012; Schnuerch and Gibbons, 2014; Schnuerch et al., 2014). FRN is suggested as a neural signature of the prediction error of behavioral results of the people and originates in an ACC (Gehring and Willoughby, 2002; Holroyd et al., 2002; Nieuwenhuis et al., 2004). When the real feedback deviates from what we expect, more negative FRN amplitude is evoked, thus the FRN can signal the performance monitoring, e.g., conflict/error detection (Kim et al., 2012; San Martín, 2012; Hauser et al., 2014; Ullsperger et al., 2014). Our result of FRN is consistent with these studies and illustrates that the larger deviation of the self-rating from the group, the more negative FRN is elicited. We suggest an early conflict detection happens when the social information (opinion of the group) is shown to the people in the situation of the crowdfunding investment decision.

Besides the FRN, a later LPP was observed in this study, with a significantly larger LPP evoked by a small conflict than the big conflict. This result is consistent with the previous studies that indicate later elaborate processing during the social information valuation (Chen et al., 2010a; Cascio et al., 2015; Schnuerch and Gibbons, 2015; Schnuerch et al., 2016). Many conformity studies with fMRI find brain regions of the ventral striatum and ventromedial prefrontal cortex activated in response to consistent social information, which indicates that people regard the alignment with a group as a social reward (Campbell-Meiklejohn et al., 2010; Zaki et al., 2011; Schnuerch and Gibbons, 2014; Cascio et al., 2015). The involvement of the vmPFC implicates the social information integration and value computing during the social information processing (Schnuerch and Gibbons, 2014; Cascio et al., 2015; Toelch and Dolan, 2015). The functional interpretation of the LPP can also reflect these neural processes. Previous studies have suggested the modulation of LPP is an indicator of the stimuli motivational significance, and hence, stimuli such as reward, arousing emotion pictures, can activate the attentional systems, resulting in the larger LPP amplitude (Hajcak and Foti, 2020). For example, LPP is more pronounced when processing attractive faces compared with unattractive faces (Thiruchselvam et al., 2016; Ma et al., 2017). Other studies find the pictures depicting pleasant/unpleasant scenes can elicit larger LPP than those depicting neutral scenes (Hajcak et al., 2006, 2013; Lin et al., 2018). These stimuli convey rewards or values that are motivationally relevant or important to the people and therefore evoke the prioritized attention to the process (Potts et al., 2006; Bloom et al., 2013). In a word, larger LPP mainly reflects the later allocation of the attention resource to selectively process the highly significant stimuli (Hajcak and Foti, 2020). In the several social influence studies, enhanced LPP is found during the processing of information that reveal own behavior or opinion of the individual is in line with or close to the group (Kim et al., 2012; Schnuerch and Gibbons, 2015; Schnuerch et al., 2016; Thiruchselvam et al., 2017). For instance, Schnuerch and Gibbons (2014) find that the information of a higher percentage of the people who agree with the choices of participants elicits lager LPP than the low agreement information, indicating agreeing with the majority is rewarding feedback and processed intensively. Another study about buying a book online illustrates that the consistent evaluation of the book by others would give the participants a positive signal, which attracts more attention and result in a lager LPP (Chen et al., 2010a). Some studies reveal that the people are more sensitive to the confirming information regarding their past choice or judgment than disconfirming information (Tarantola et al., 2017; Kappes et al., 2019), implying that small conflict social information might be more valuable to people than the big conflict. In our study, when the willingness-to-invest of the participants is close to the group (small conflict), it provides stronger social proof for own judgments of the participants and might be regarded as a reward signal, which is of the higher subjective value and more motivationally significant, and therefore leads to a larger LPP than the big conflict. In conclusion, LPP can be used to track the significance level of the social information and corresponding attention allocated to the valuation process of this information. Besides, the LPP difference can account for the conformity behaviors difference elicited by the small and big conflicts. Specifically, when the own ratings of the investors are similar with the ratings of the group, they are less motivated to adjust their behavior since it is considered as a reward for them. In contrast, when their own ratings are far from the group, the error signal is stronger, then the motivation to change their behavior becomes more significant. Our results also contribute to the theoretical interpretation of the reinforcement learning theory for the social influence (Klucharev et al., 2009; Campbell-Meiklejohn et al., 2010; Shestakova et al., 2013; Schnuerch and Gibbons, 2014).

This study also discovers the effect of confidence on the social information evaluation, but only at the later stage. There was not difference in an FRN between low- and high-confidence conditions while larger LPP was evoked in low confidence. Besides, the LPP difference between small and large conflicts was more significant in the low-confidence condition than that in the high-confidence condition. These results reveal that initial confidence mainly affects the later valuation of the social information rather than the early conflict detection and the uncertain experience of the people could amplify the perception difference when the participants evaluate the two kinds of social information. It is suggested both the individual information and observed social information are integrated to reach the decision and the weights on each source of the information depend on several factors, such as personal predispositions and stimulus ambiguity, therefore allowing for the different magnitudes of the conformity behaviors (Toelch and Dolan, 2015; Park et al., 2017). For example, a study finds that individual information is over proportionally weighted, particularly under low uncertainty derived from the prior high-individual accuracy (Toelch et al., 2014). Social information is more likely used when uncertainty increases (Toelch et al., 2009). Several studies experimentally reduce the confidence of an individual and find people who would increase reliance on social information (Estes and Felker, 2012; Morgan et al., 2012; Cross et al., 2017). Other neural studies indicate that confidence influences the selective neural gating for external information, and holding high confidence makes choice-inconsistent information abolished, which finally leads to decreasing likelihood of changes of the mind (Atiya et al., 2019; Rollwage et al., 2020). The aforementioned studies imply that the social information, especially consistent information with self, is more weighted in low confidence. Besides, prior studies indicate that uncertainty could enhance motivated attention (Anselme, 2010). Therefore, this disproportionate reliance on social information can be reflected by the LPP deflection in this study. Larger LPP in low confidence indicates that the social information is more important to the participants and attracts more attention. The confidence effect in our study indicates the adaptive behavior of humans to increase their payoff when making investment decisions, and confidence can modulate learning the value of behavior of others.

There are two limitations of this study that might be remedied by future work. First, since the sample of our participants was predominately males, it should be cautious to claim that our findings hold across genders. Although many previous studies have demonstrated similar effect of the social influence with solely male sample (e.g., Zaki et al., 2011), solely female sample (e.g., Klucharev et al., 2009; Shestakova et al., 2013), and mixed gender sample (e.g., Campbell-Meiklejohn et al., 2010; Huang et al., 2014; Nook and Zaki, 2015; Schnuerch and Gibbons, 2015), suggesting that the current findings may generalize across genders, future research using the balanced proportion of gender in the sample are still required to provide more evidence for our findings. Second, this study neglected the collection of the subjective value of the participants about the different conflicts, which could have provided more complement and support to the LPP results in this study.

## Conclusion

This study applied the event-related potentials method to examine the neural mechanisms of social influences and their interaction with the confidence in crowdfunding investment. The results demonstrate the typical conformity behavior and underlying neural responses to the social information consisting of conflict detection and valuation. People are likely to change their opinions in line with the group and are sensitive to the conflict between their own opinions and the group, with the big conflict more likely detected while small conflict evaluated more positive. Furthermore, confidence is verified to affect the social influence both behaviorally and neurologically. Low confidence in the decision leads to more conformity behaviors and increases more reliance on social information. This study extends the existing literature on social influence and confidence, by deepening our understanding of their relationship. The fluctuant confidence in the decision might be implicated as a strong predictor of susceptibility to social influence.

## Data Availability Statement

The raw data supporting the conclusions of this article will be made available by the authors, without undue reservation.

## Ethics Statement

This study was reviewed and approved by the Ethics Committee of Neuromanagement Laboratory in Zhejiang University. The participants provided their written informed consent to participate in this study.

## Author Contributions

JZ: research conception, experiment design, data collection, data analysis, and writing the initial and final draft. LH: experimental design, data analysis, and writing the initial and final draft. LL: data collection, data analysis, and review the final draft. QS: research conception, experiment design, and data analysis. LW: research conception, experiment design, and review and writing the final draft. All authors contributed to the article and approved the submitted version.

## Conflict of Interest

The authors declare that the research was conducted in the absence of any commercial or financial relationships that could be construed as a potential conflict of interest.

## Publisher’s Note

All claims expressed in this article are solely those of the authors and do not necessarily represent those of their affiliated organizations, or those of the publisher, the editors and the reviewers. Any product that may be evaluated in this article, or claim that may be made by its manufacturer, is not guaranteed or endorsed by the publisher.
